# Natural Product Driven Activation of UCP1 and Tumor Metabolic Suppression: Integrating Thermogenic Nutrient Competition with Cancer Metabolic Reprogramming

**DOI:** 10.3390/biom16010090

**Published:** 2026-01-06

**Authors:** Dong Oh Moon

**Affiliations:** Department of Biology Education, Daegu University, 201, Daegudae-ro, Gyeongsan-si 38453, Gyeongsangbuk-do, Republic of Korea; domoon@daegu.ac.kr

**Keywords:** UCP1, PRDM16, natural products

## Abstract

Metabolic reprogramming allows cancer cells to proliferate rapidly, survive nutrient limitation, and resist stress, making tumor metabolism an important therapeutic target. However, pharmacological inhibition of metabolic enzymes often causes systemic toxicity and compensatory pathway activation. To overcome these limitations, recent studies have highlighted an alternative host-centered strategy based on increasing systemic energy expenditure. Recent studies highlight an alternative strategy in which the host increases energy expenditure through uncoupling protein 1 (UCP1) dependent thermogenesis, thereby lowering systemic glucose, fatty acid, and nucleotide availability for tumors. Engineered beige adipocytes overexpressing UCP1, PR domain-containing protein 16 (PRDM16), or peroxisome proliferator–activated receptor gamma coactivator 1 alpha (PPARGC1A/PGC1A) suppress tumor growth through nutrient competition, suggesting that activating endogenous UCP1 may provide a non-genetic and physiologically aligned anticancer approach. Building on this concept, natural products such as polyphenols, terpenoids, alkaloids, and carotenoids have emerged as promising UCP1 activators that stimulate beige and brown adipocyte thermogenesis through pathways involving AMP-activated protein kinase (AMPK), sirtuin 1 (SIRT1), PGC1A, PRDM16, and mitochondrial biogenesis. In parallel, computational studies further indicate that several plant-derived compounds bind directly to the central cavity of UCP1 with high affinity, offering structural support for their thermogenic action. Importantly, many of these compounds also inhibit cancer cell intrinsic metabolism by reducing glycolysis, oxidative phosphorylation, lipid synthesis, and amino acid dependent anaplerosis. This review integrates UCP1 biology, natural product mediated thermogenesis, molecular docking evidence, and tumor metabolic suppression, proposing a unified framework in which natural compounds impose coordinated metabolic pressure on cancer through both adipocyte-driven nutrient competition and direct inhibition of tumor metabolism.

## 1. Introduction

Metabolic reprogramming has emerged as a fundamental hallmark of cancer, enabling malignant cells to sustain accelerated proliferation, resist cell death, and adapt to nutrient restricted microenvironments. Tumors rely heavily on glycolysis, fatty acid oxidation, and nucleotide metabolism to maintain bioenergetic and biosynthetic demands, a phenomenon broadly described as metabolic plasticity [1,2]. Given this dependency, metabolic interference has long been considered a potential anticancer strategy; however, direct inhibition of tumor metabolic enzymes frequently leads to systemic toxicity, compensatory pathway activation, or limited therapeutic selectivity. These limitations have stimulated interest in systemic metabolic interventions that deprive tumors of nutrients not by targeting the cancer cell directly, but by modifying whole body metabolism. This perspective reframes cancer metabolic reprogramming as a process that may be indirectly exploited by altering host energy utilization rather than directly inhibiting tumor metabolic enzymes.

Within this conceptual shift, the idea of nutrient competition that host tissues can consume essential fuels faster than a tumor has gained attention. A series of recent studies demonstrate that enhancing energy expenditure in peripheral tissues can reduce the availability of glucose, fatty acids, and nucleotides to tumors, thereby suppressing tumor growth through metabolic starvation. Brown adipose tissue (BAT) and beige adipocytes are uniquely positioned at the center of this concept, owing to their remarkable capacity for thermogenic metabolism, a process governed by the mitochondrial proton transporter UCP1 [3,4]. Classic physiological studies established BAT as a major thermogenic organ in mammals, with UCP1 serving as the essential molecular effector that dissipates mitochondrial proton gradients as heat [5,6,7].

However, despite compelling evidence that UCP1-positive brown and beige adipocytes can suppress tumor growth through nutrient competition, no study has yet demonstrated that natural product–driven activation of UCP1 thermogenesis is sufficient to induce tumor suppression via this mechanism. This unresolved question represents a critical link between systemic metabolic reprogramming and pharmacological intervention that remains unexplored.

**Figure 1:**
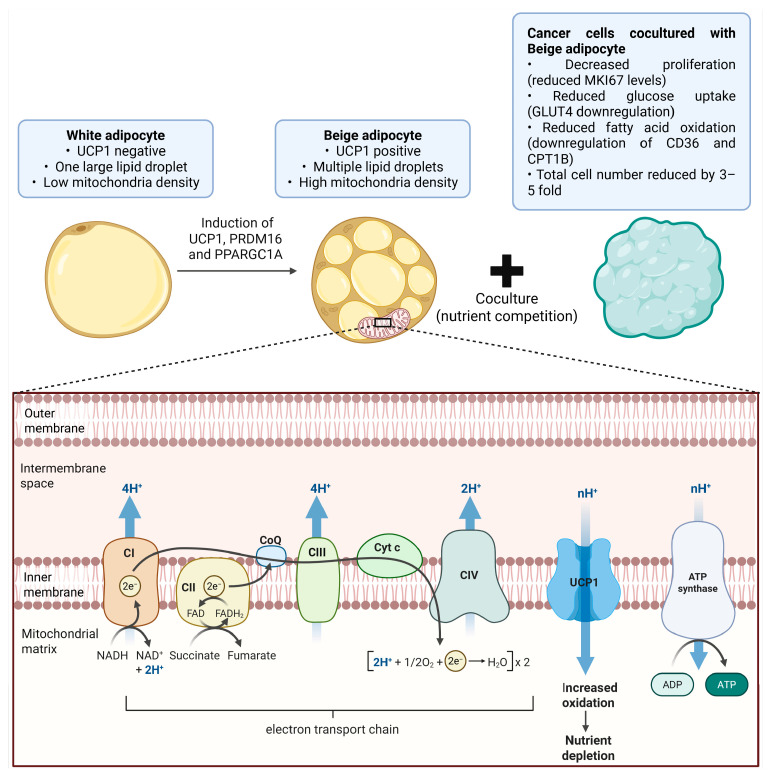
Beige Adipocyte–Driven UCP1 Activation Enhances Mitochondrial Oxidation and Suppresses Cancer Cell Metabolism Through Nutrient Competition. White adipocytes are characterized by a unilocular lipid droplet, low mitochondrial density, and an absence of UCP1 expression. Upon induction of UCP1, PRDM16, and *PPARGC1A*, these cells differentiate into beige adipocytes, which contain multiple lipid droplets, a high density of mitochondria, and robust UCP1 expression. When cocultured with cancer cells, beige adipocytes inhibit tumor cell growth, as demonstrated by decreased proliferation (reduced MKI67 levels), reduced glucose uptake (GLUT4 downregulation), reduced fatty acid oxidation (CD36 and CPT1B downregulation), and an overall 3–5-fold reduction in total cancer cell number, primarily through nutrient competition. Representative coculture studies report a ~2–3-fold increase in glucose and fatty acid uptake by beige adipocytes, accompanied by an approximate ~40–60% reduction in tumor cell proliferation and ATP availability. The lower panel illustrates the mitochondrial electron transport chain in beige adipocytes. Complexes I, III, and IV pump protons into the intermembrane space, establishing the electrochemical gradient required for ATP synthesis. UCP1 mediates proton leak across the inner mitochondrial membrane, dissipating the proton motive force as heat and driving increased substrate oxidation. This elevated oxidative metabolism depletes extracellular nutrients, contributing to metabolic starvation of cocultured cancer cells. Together, these processes highlight the metabolic interplay by which UCP1 positive beige adipocytes exert antitumor effects. The cartoon in Figure 1 was created with BioRender.com (https://app.biorender.com, accessed on 1 August 2025). Abbreviations: UCP1, uncoupling protein 1; PRDM16, PR domain–containing protein 16; *PPARGC1A*/PGC1α, peroxisome proliferator–activated receptor gamma coactivator 1α; MKI67, marker of proliferation Ki-67; GLUT4, glucose transporter 4; CD36, fatty acid translocase; CPT1B, carnitine palmitoyltransferase 1B.

Two landmark studies recently demonstrated that UCP1 dependent thermogenesis can suppress tumor progression. Implantation of engineered beige adipocytes overexpressing UCP1 or browning regulators significantly reduced tumor growth in multiple cancer models, primarily by competing with tumors for glucose, fatty acids, and nucleosides [8,9]. In particular, Nguyen and colleagues used CRISPR activation to upregulate *UCP1*, *PRDM16*, or *PPARGC1A* in human white adipocytes and adipose organoids, which converted them into highly oxidative beige adipocytes with increased glucose uptake, fatty acid oxidation, and mitochondrial respiration [8]. When these CRISPRa engineered adipocytes were cocultured with cancer cells or implanted adjacent to xenografts derived from breast, colon, pancreatic, and prostate tumors, they markedly reduced tumor growth, hypoxia, and angiogenesis by outcompeting tumors for metabolic substrates [8]. This adipose manipulation transplantation strategy illustrates how enforcing a stable thermogenic program in adipocytes can transform them into metabolic sinks that starve tumors of nutrients, and this concept is summarized schematically in Figure 1.

An important implication of these findings is that robust transcriptional induction of *UCP1*, *PRDM16*, and *PPARGC1A* may be more effective than acute pharmacological activation of pre-existing protein pools. UCP1 expression sets the thermogenic capacity of adipose depots, while PRDM16 and PGC1α, the protein encoded by *PPARGC1A*, act as master regulators of the beige and brown adipocyte gene program and mitochondrial biogenesis, and sustained expression of these factors is required to maintain a thermogenic fate in adipocytes [10,11]. In contrast, transient allosteric activators or post translational modifiers of UCP1 can increase proton leak only as long as the protein is present, without expanding the pool of UCP1 positive mitochondria or enforcing a stable beige transcriptional identity. Moreover, PGC1α and related coactivators are also coopted by tumor cells to support oxidative phosphorylation, stress resistance, and metastatic fitness [12], which suggests that selectively driving UCP1, PRDM16, and *PPARGC1A* expression in adipose tissue, rather than globally activating their downstream pathways, may be critical for safe and effective metabolic cancer therapies.

Natural products including polyphenols, terpenoids, alkaloids, and carotenoids have emerged as potent modulators of UCP1 and its upstream signaling pathways. Compounds such as resveratrol [13], capsaicin and curcumin [14] activate thermogenic programs through AMPK SIRT1 PGC1α signaling, β3-adrenergic pathways, PRDM16 stabilization, and mitochondrial biogenesis. These bioactive molecules not only drive browning of white adipose tissue but also exert independent anticancer effects, suggesting a unique opportunity to integrate natural product pharmacology with UCP1 centered metabolic cancer therapy.

A key advantage of natural product–based thermogenic competition models is their potential to activate endogenous UCP1 programs without genetic modification, enabling physiologically aligned and potentially safer strategies to increase systemic energy expenditure. Unlike engineered adipocytes, natural compounds can be administered orally or systemically, may act on multiple adipose depots simultaneously, and can induce sustained thermogenic transcriptional programs that reduce nutrient availability to tumors while minimizing off-target toxicity. Furthermore, many thermogenic natural products simultaneously suppress tumor metabolism directly by inhibiting glycolysis, oxidative phosphorylation, or lipid biosynthesis creating a dual mechanism in which adipocytes and cancer cells are both metabolically constrained.

This review synthesizes current evidence for UCP1 biology, its regulation by natural compounds, and the emerging concept of UCP1-centered nutrient competition as a promising metabolic anticancer strategy. In addition, natural products that induce or activate UCP1, PRDM16, and PPARGC1A in brown and beige adipocytes are summarized, and compounds that directly suppress tumor metabolism, including those targeting glycolysis, mitochondrial function, and lipid metabolism, are highlighted. By integrating thermogenic adipocyte biology with natural-product pharmacology, future opportunities for developing systemic metabolic interventions against cancer based on targeted induction of the UCP1–PRDM16–PGC1α axis are outlined. Within this metabolic framework, UCP1 emerges as a central regulator of systemic energy expenditure with direct implications for nutrient availability to tumors. The literature for this review was identified through systematic searches of PubMed and Web of Science databases, covering studies published between January 2000 and March 2025. Boolean search terms included combinations of “UCP1” AND “thermogenesis” AND “cancer,” “natural products” AND “UCP1,” “brown adipose tissue” OR “beige adipocytes,” and “resveratrol” OR “sirtuins” AND “metabolic reprogramming.” Reference lists of relevant articles were also manually screened to identify additional pertinent studies.

## 2. UCP1 Biology and Thermogenic Mechanisms

UCP1 represents a unique metabolic node that links mitochondrial energy dissipation to whole-body nutrient utilization. By increasing proton leak–driven respiration, UCP1 promotes accelerated oxidation of glucose- and lipid-derived substrates, thereby shifting cellular metabolism toward catabolic energy consumption rather than anabolic storage. UCP1 is a mitochondrial inner membrane protein that functions as the primary molecular effector of non-shivering thermogenesis in brown and beige adipocytes. Structurally, UCP1 belongs to the mitochondrial solute carrier family and contains six transmembrane α-helices that form a proton transport channel within the inner mitochondrial membrane [5]. Unlike classical oxidative phosphorylation where proton flow through ATP synthase produces ATP, UCP1 dissipates the proton gradient as heat, thereby uncoupling substrate oxidation from ATP generation. This proton leak–driven thermogenesis dramatically increases mitochondrial respiration and whole-body energy expenditure [5,7].

UCP1 expression is markedly enriched in BAT, a thermogenic organ characterized by multilocular lipid droplets, dense mitochondrial content, extensive sympathetic innervation, and a gene program driven by transcriptional regulators such as PRDM16, PGC1α, and early B cell factor 2 (EBF2) [6]. In contrast, white adipose tissue (WAT) primarily functions as an energy storage depot, containing unilocular adipocytes with few mitochondria and negligible UCP1 expression. Beige adipocytes, which emerge within WAT upon cold exposure, β-adrenergic stimulation, exercise-induced myokines, or certain natural compounds, display an inducible thermogenic phenotype. Functionally, beige and brown adipocytes oxidize fatty acids released from intracellular lipid droplets while simultaneously increasing glucose uptake to sustain elevated mitochondrial flux, thereby directly influencing systemic carbohydrate and lipid homeostasis. Unlike classical BAT, beige adipocytes can reversibly switch between thermogenic and energy-storing states depending on environmental or hormonal cues [6].

Genetic ablation of UCP1 has provided key insights into the biological relevance of thermogenesis. UCP1 knockout mice exhibit profound cold sensitivity, impaired adaptive thermogenesis, and an inability to increase energy expenditure in response to high-calorie feeding [7,15]. Although UCP1-deficient mice do not always develop obesity under standard conditions, they show reduced metabolic flexibility and an altered systemic nutrient utilization pattern. These alterations reflect a shift toward energy conservation and anabolic storage, highlighting the role of UCP1 in maintaining catabolic metabolic tone at the organismal level. Importantly, recent cancer studies revealed that UCP1 is essential for thermogenesis-driven metabolic tumor suppression. Cold-induced BAT activation fails to inhibit tumor growth in UCP1-deficient mice, demonstrating that UCP1-mediated nutrient competition rather than other cold-associated physiological changes is the critical effector suppressing tumor progression [9]. Similarly, implantation of engineered beige adipocytes lacking UCP1 abolishes their tumor-suppressive capacity, underscoring UCP1 as an indispensable metabolic driver of anticancer energy expenditure [8].

Although previous reviews have broadly summarized the thermogenic roles of UCP1 and its relevance to obesity management and neurodegenerative conditions [16], these works have not addressed the emerging concept that UCP1 driven beige and brown adipocytes can directly influence tumor progression through systemic metabolic remodeling. Existing reviews primarily focus on natural or synthetic thermogenic agents in the context of weight reduction, energy expenditure or neuronal protection, without integrating the recent evidence demonstrating that thermogenic adipocytes can function as metabolic sinks that restrict tumor nutrient availability. In contrast, the present review synthesizes UCP1 signaling pathways with natural product-based activation and highlights their potential in suppressing tumor metabolic flexibility, glycolytic dependency and nutrient acquisition. By positioning UCP1 at the intersection of nutrient oxidation, systemic metabolic reprogramming, and tumor energy supply, this review establishes a coherent framework linking thermogenic biology to cancer metabolism. This cancer-oriented perspective has not been previously consolidated, particularly in light of recent Nature studies showing that cold induced or engineered beige adipocytes exert robust antitumor effects through nutrient competition. By integrating thermogenic biology, natural compound pharmacology and cancer metabolism, this review fills a distinct gap in the literature and proposes UCP1 centered thermogenic activation as a novel metabolic anticancer strategy.

## 3. Signaling Pathways Regulating UCP1 Expression

**Figure 2:**
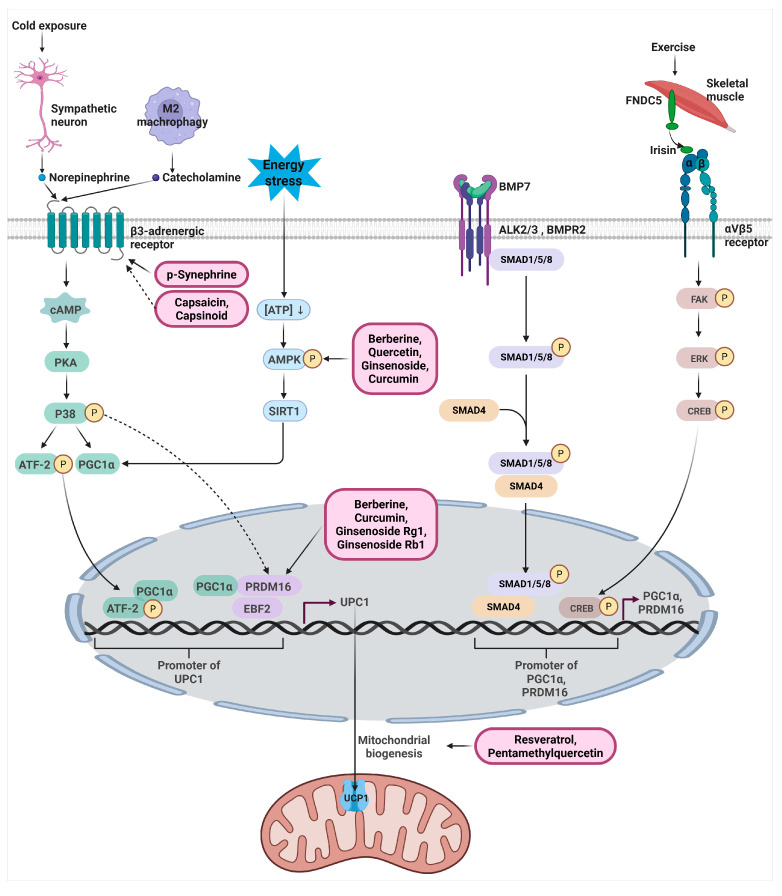
Integrated signaling pathways regulating UCP1 transcription in brown and beige adipocytes. This schematic illustrates the major thermogenic signaling pathways that converge on the transcriptional activation of UCP1 in brown and beige adipocytes. Cold exposure triggers sympathetic neuron–derived norepinephrine release, which activates β3 adrenergic receptors and initiates the cAMP–PKA–p38 MAPK cascade, leading to phosphorylation and activation of ATF2 and increased PGC1α expression. Energy stress elevates AMPK activity, promoting SIRT1 mediated deacetylation and activation of PGC1α, which cooperates with PRDM16 and EBF2 to induce UCP1 transcription. BMP7 binding to ALK2/3–BMPR2 receptors activates SMAD1/5/8 phosphorylation and nuclear translocation with SMAD4, enhancing PRDM16 and PGC1α promoter activity. Exercise induced irisin, cleaved from skeletal muscle FNDC5, binds integrin αVβ5 on adipocytes and activates the FAK–ERK–CREB pathway, further promoting PGC1α and PRDM16 expression. p-Synephrine, capsaicin, and capsinoids enhance thermogenesis by mimicking or indirectly stimulating β3-adrenergic signaling, resulting in increased expression of UCP1, PRDM16, and PGC1α and improved browning and metabolic activity. Berberine, quercetin, ginsenoside Rg1, ginsenoside Rb1, and curcumin activate AMPK signaling to induce UCP1 expression, promote browning, and enhance mitochondrial function in adipose tissue. Berberine, curcumin, ginsenoside Rg1, and ginsenoside Rb1 also strengthen PRDM16 centered transcriptional programs by elevating PRDM16 and PGC1α expression or by improving chromatin accessibility at thermogenic loci. Resveratrol and PMQ stimulate mitochondrial biogenesis through activation of the SIRT1–PGC1α axis, increasing mitochondrial DNA, oxidative capacity, and UCP1 levels, thereby promoting the formation of mitochondria-rich beige adipocytes. Direct signaling events are shown with solid arrows, whereas dotted arrows indicate indirect or second order regulatory effects. The cartoon in Figure 2 was created with BioRender.com (https://app.biorender.com, accessed on 2 September 2025).

UCP1 expression is regulated through multiple convergent signaling pathways that integrate sympathetic input, metabolic sensing, transcriptional programs and endocrine cues. These pathways collectively define the thermogenic competence of brown and beige adipocytes and determine systemic energy expenditure. In recent years it has become clear that these pathways not only act independently but also exhibit extensive cross talk at the levels of chromatin remodeling, mitochondrial dynamics and substrate oxidation, and therefore UCP1 regulation should be viewed as the coordinated output of a multi layered signaling architecture rather than a simple linear cascade. The signaling pathways regulating UCP1 expression are illustrated in Figure 2.

### 3.1. β3-Adrenergic Signaling Pathway

Cold exposure activates sympathetic neurons that release norepinephrine onto adipocytes. Norepinephrine binds to β3-adrenergic receptors and elevates intracellular cAMP, leading to activation of protein kinase A (PKA) [17]. PKA phosphorylates and activates p38 MAP kinase, a central signaling node that drives the transcriptional thermogenic program. Activated p38 MAPK increases the expression and activity of ATF-2 and PGC1α through cAMP responsive elements and thermogenic enhancers, and these factors subsequently translocate to the nucleus where they bind UCP1 regulatory elements to directly promote UCP1 transcription [18,19,20]. In addition, p38 MAPK recruits histone acetyltransferases that increase chromatin accessibility at the UCP1 enhancer, further facilitating transcription factor engagement. Pharmacological inhibition of p38 MAPK suppresses UCP1 induction in both brown and beige adipocytes, highlighting its essential role in adrenergic thermogenesis [18,21]. Beyond transcriptional control, β3-adrenergic signaling stimulates lipolysis, generating fatty acids that fuel mitochondrial oxidation and allosterically activate UCP1, thereby coupling metabolic substrate flux with transcriptional induction of the thermogenic machinery. Cardiac natriuretic peptides can also activate p38 MAPK independently of β-adrenergic receptors, underscoring the conserved nature of this signaling module across multiple hormonal inputs [22].

### 3.2. AMPK-SIRT1-PGC1α Metabolic Signaling Pathway

The AMPK-SIRT1- PGC1α pathway functions as a metabolic sensor linking nutrient availability to thermogenic programming. AMPK is activated by increased AMP to ATP ratios and phosphorylates downstream targets that enhance mitochondrial function. AMPK activation increases SIRT1 activity, which deacetylates and activates PGC1α, resulting in mitochondrial biogenesis and UCP1 induction [23]. Beyond PGC1α activation, AMPK facilitates fatty acid oxidation through phosphorylation of acetyl CoA carboxylase, thereby ensuring efficient substrate flux toward mitochondria during thermogenic activation. Loss of function studies show that AMPK deficient adipocytes exhibit impaired thermogenesis and diminished UCP1 expression, confirming that AMPK signaling is necessary for beige adipocyte recruitment under metabolic stress [24]. Moreover, AMPK signaling influences autophagy dependent mitochondrial quality control, which is increasingly recognized as an important determinant of sustained UCP1 mediated thermogenesis.

### 3.3. PRDM16 -PGC1α-EBF2 Transcriptional Pathway

The transcriptional foundation of thermogenic adipocyte identity is orchestrated by the PRDM16 PGC1α EBF2 pathway. PRDM16 drives brown adipocyte lineage commitment and activates thermogenic genes, working cooperatively with PGC1α to stimulate UCP1 expression [25]. PRDM16 interacts with a network of chromatin modifiers including histone demethylases and coactivator complexes, which stabilize thermogenic enhancers and suppress white adipocyte gene programs. EBF2 binds brown adipocyte enhancers and recruits chromatin remodeling complexes that maintain an open chromatin state at UCP1 regulatory regions [26]. Perturbation of PRDM16 or EBF2 results in loss of beige adipocyte conversion and diminished UCP1 expression, demonstrating the essential role of this transcriptional pathway [27]. Notably this pathway integrates signals from adrenergic and AMPK dependent pathways through convergence on PGC1α, indicating that thermogenic transcriptional identity and metabolic activation are inseparable components of UCP1 regulation.

### 3.4. Endocrine and Paracrine Thermogenic Pathways (BMP7, Irisin, METRNL)

Thermogenesis is also regulated by endocrine factors that act independently of sympathetic input. Among these, bone morphogenetic protein 7 (BMP7) functions as a potent systemic signal that directly promotes the differentiation and thermogenic programming of brown and beige adipocytes. BMP7 binds to type I (ALK2 or ALK3) and type II (BMPR2) BMP receptors on preadipocytes, triggering phosphorylation of Smad1, Smad5 and Smad8. The activated Smad complex forms a heterotrimer with Smad4 and translocates into the nucleus, where it induces the expression of brown-fat–selective transcriptional regulators including PRDM16, PGC1α and Ucp1 itself. Through this Smad-dependent transcriptional program, BMP7 enhances mitochondrial biogenesis, increases oxidative capacity and promotes a full thermogenic gene expression profile. Notably, these effects occur even in the absence of β-adrenergic stimulation, demonstrating that BMP7 provides an alternative, adrenergic-independent mechanism for initiating and sustaining UCP1-driven thermogenesis [28].

Exercise increases PGC1α activity in skeletal muscle, which enhances the expression of fibronectin type III domain containing protein 5, also known as Fibronectin type III domain–containing protein 5 (*FNDC5*). FNDC5 is produced as a membrane anchored protein and is then cleaved at the cell surface to release its extracellular portion as irisin into the bloodstream. Irisin acts as an endocrine myokine that binds to the integrin αVβ5 receptor complex on white adipocytes, activating intracellular signaling pathways that include focal adhesion kinase, ERK, and p38 MAPK. These signals drive the conversion of white adipocytes into a thermogenic state. This signaling cascade promotes the expression of important transcriptional regulators such as PGC1α, PRDM16, and EBF2. These factors increase chromatin accessibility at thermogenic gene regions and enhance the assembly of transcriptional machinery at the UCP1 promoter. As a result, irisin induces the appearance of multilocular and mitochondria rich beige adipocytes within white adipose tissue. This leads to increased UCP1 expression, higher fatty acid oxidation, elevated mitochondrial activity, and a general improvement in thermogenic capacity [29].

Meteorin like protein is secreted from skeletal muscle and subcutaneous adipose tissue in response to exercise, cold exposure, and adrenergic stimulation, and it acts primarily on type 2 immune cells rather than directly on adipocytes. Meteorin-like protein (METRNL) activates eosinophils through yet unidentified surface receptors, leading to interleukin 4 and interleukin 13 release, which polarize macrophages toward the M2 phenotype and create an immune derived signaling environment that enhances PGC1α and UCP1 transcription in emerging beige adipocytes. [30]. These endocrine pathways highlight the integration of skeletal muscle, immune cells and adipose tissue in the systemic regulation of thermogenesis.

## 4. Natural Products That Upregulate UCP1 Expression and Thermogenic Programming

Given the central role of UCP1 in regulating thermogenic energy expenditure, identifying pharmacological strategies that activate UCP1 without genetic manipulation has become a key focus. A wide range of plant derived compounds enhance UCP1 expression by acting on the signaling axes described above, including AMPK-SIRT1-PGC1α, β3-adrenergic pathways, PRDM16 centered transcriptional programs and mitochondrial biogenesis. Below, representative natural products are organized according to their dominant mechanism, with emphasis on transcriptional induction and thermogenic programming of UCP1 in brown and beige adipocytes. Evidence related to direct activation of UCP1 at the protein level is addressed separately in Section 6.

### 4.1. Natural Products That Mimic β3-Adrenergic Receptor Activation

p-Synephrine, the major proto-alkaloid of Citrus aurantium, acts as a β3-adrenergic-like compound in adipose tissue, leading to increased expression of thermogenic genes including UCP1, PRDM16 and PGC-1α in brown and beige adipocytes in vivo. These changes are associated with improved brown adipose tissue morphology and metabolic parameters in obesity models [31]. Capsaicin, a TRPV1 agonist found in chili peppers, has been shown to promote adipocyte browning by activating sensory neurons and the sympathetic nervous system which in turn stimulates β3-adrenergic signaling in adipose tissue. In rodent models and human cell systems, capsaicin treatment increased UCP1 expression, mitochondrial biogenesis, and oxygen consumption in white or brown adipocytes, indicating enhanced thermogenic capacity [32]. Capsinoids, non-pungent analogues of capsaicin, activate TRPV1 receptors in the gut and stimulate sympathetic output. In human human interventional study, daily ingestion of 9 mg capsinoids for 6 weeks increased supraclavicular BAT vascular density and showed a trend toward higher resting energy expenditure, indicating activation of UCP1-positive adipose depots [33].

### 4.2. AMPK Activating Natural Products

Berberine is one of the best characterized phytochemicals that activate AMPK signaling. In diet induced obese mice, berberine increased AMPK phosphorylation in white and brown adipose tissue, induced UCP1 and other thermogenic genes, promoted browning of inguinal white adipose depots and increased oxygen consumption, while genetic or pharmacologic inhibition of AMPK blunted these effects [34]. In a human interventional study in patients with non-alcoholic fatty liver disease, one month of berberine treatment increased brown adipose tissue mass and activity and improved insulin sensitivity, and mechanistic work in parallel showed that berberine activates AMPK and upregulates PRDM16 and UCP1 in adipocytes [35].

Quercetin also enhances UCP1 through an AMPK dependent mechanism. Dietary quercetin supplementation in high fat diet fed mice increased UCP1 protein in white and brown adipose tissue and elevated sympathetic activity, while in vitro experiments showed that quercetin activated AMPK and induced a thermogenic gene program [36]. Ginsenoside Rg1 from ginseng induces browning of subcutaneous white adipocytes through AMPK activation; Rg1 treatment increased UCP1, PGC1α and mitochondrial biogenesis markers in 3T3 L1 adipocytes and in white adipose tissue of treated mice [37]. Black ginseng extract and the ginsenoside Rb1 similarly promote AMPK activation, increase UCP1 and PRDM16 expression and stimulate browning in 3T3 L1 cells and perirenal white adipose tissue [38].

Curcumin has repeatedly been shown to increase UCP1 in vitro and in vivo. Curcumin induced a brown fat like phenotype in 3T3 L1 and primary white adipocytes with robust upregulation of UCP1 and other brown markers [39], and dietary curcumin intervention in obese mice reduced white adipose inflammation while increasing UCP1 protein in brown adipose tissue [40].

### 4.3. Natural Products That Enhance PRDM16 Centered Transcriptional Programs

Several natural compounds reinforce PRDM16-driven thermogenic transcription, either by increasing PRDM16 expression or by improving its chromatin regulatory environment. In a detailed mechanistic study, berberine increased transcription of Prdm16 in brown and beige adipose tissue by promoting active DNA demethylation at the Prdm16 promoter through an AMPK dependent increase in alpha ketoglutarate, leading to higher UCP1 expression and greater recruitment of brown adipose tissue in humans and mice [35]. Curcumin not only induces UCP1 but also increases mRNA and protein levels of PRDM16, PGC1α and PPAR gamma in white adipocytes and subcutaneous adipose tissue, supporting assembly of a thermogenic transcriptional complex at UCP1 regulatory regions [39,40]. Multiple ginsenosides including Rg1 and Rb1 increase PRDM16 and PGC1α together with UCP1 in cultured adipocytes and white adipose tissue, consistent with a coordinated activation of the PRDM16 PGC1α axis [37,38].

### 4.4. Natural Products That Enhance BMP7–SMAD Pathway or the FNDC5–Irisin Pathway

At present, no natural products have been conclusively demonstrated to induce UCP1 expression through direct activation of the BMP7–SMAD or FNDC5–irisin pathways. However, several preclinical studies have explored the potential of natural compounds, including flavonoids and glycosides, to modulate downstream components of these endocrine thermogenic pathways. In particular, selected phytochemicals have been reported to influence SMAD-dependent transcriptional activity or integrin-mediated signaling that overlaps with BMP7 or FNDC5–irisin signaling cascades in adipose or muscle-related experimental contexts.

Although several phytochemicals have been proposed as potential modulators of SMAD-dependent or integrin-mediated signaling, direct experimental evidence linking these compounds to UCP1 induction via endocrine thermogenic pathways remains limited and largely preclinical. While these observations suggest a possible indirect link between certain natural products and UCP1 regulation through endocrine mechanisms, none of the available studies have established a causal sequence connecting natural compound treatment to BMP7- or irisin-dependent UCP1 induction in brown or beige adipocytes.

Accordingly, systematic and targeted screening of candidate natural compounds will be required to determine whether specific phytochemicals can engage BMP7–SMAD or FNDC5–irisin signaling in a manner sufficient to drive thermogenic programming and UCP1 expression. Such efforts may help clarify whether these endocrine pathways represent viable but currently underexplored mechanisms for natural product-mediated activation of adipose thermogenesis, rather than excluding them based on the present lack of definitive evidence.

### 4.5. Natural Products That Enhance Mitochondrial Biogenesis and Thermogenic Remodeling

A subset of natural compounds enhances UCP1 expression by stimulating mitochondrial biogenesis, a process essential for establishing and maintaining functional thermogenic adipocytes. Resveratrol, a polyphenolic stilbene, is the most widely studied natural compound in this category. Mechanistic work demonstrates that resveratrol activates the SIRT1–PGC1α axis, leading to robust increases in mitochondrial gene expression, mitochondrial DNA content, oxidative phosphorylation capacity, and overall mitochondrial function [41]. Beyond its role in thermogenic programming, accumulating evidence indicates that resveratrol-mediated activation of sirtuins, particularly SIRT1, exerts broad anti-inflammatory and cardiometabolic effects. Recent studies demonstrate that SIRT1 activation by resveratrol suppresses chronic inflammation through modulation of NF-κB signaling, improves mitochondrial homeostasis, and attenuates oxidative stress, thereby contributing to cardiovascular protection and systemic metabolic health. Importantly, emerging translational and clinical data suggest that these SIRT1-dependent anti-inflammatory and metabolic effects may extend to cancer-relevant contexts, where inflammation and metabolic dysregulation are key drivers of tumor progression. In addition, the methylated flavonoid pentamethylquercetin (PMQ) enhances mitochondrial biogenesis and functional thermogenesis. Experimental studies show that PMQ increases PGC1α expression, stimulates mitochondrial oxidative capacity, elevates oxygen consumption rate, and significantly upregulates UCP1 in both 3T3-L1 adipocytes and high-fat diet–fed mice [42]. These findings indicate that PMQ supports the formation of mitochondria-rich beige adipocytes with enhanced thermogenic potential. Natural products that upregulate UCP1 expression and thermogenic programming are summarized in Table 1 and Figure 2.

## 5. Structural Basis of UCP1 Regulation

Recent structural analysis has clarified the molecular basis for purine nucleotide inhibition of UCP1. The cryo EM structure of human UCP1 bound to GTP demonstrates how nucleotide binding locks the protein in a conformation that prevents proton conduction [43]. UCP1 normally conducts proton leak to generate heat, but this activity is suppressed by purine nucleotides, whereas free fatty acids activate the protein by releasing this inhibition [44].

UCP1 exhibits the characteristic architecture of the SLC25 mitochondrial carrier family, consisting of six transmembrane helices arranged into three homologous domains [45]. These domains form a central aqueous cavity controlled by cytoplasmic and matrix gates, similar to the mechanism described for the ADP ATP carrier [46,47]. In the GTP bound conformation, UCP1 is trapped in a cytoplasmic open state, while the matrix gate is firmly closed by a network of ionic interactions that include D35, K38, E135, K138, D234, and K237 [43]. This arrangement establishes an insulating barrier of approximately ten angstroms on the matrix side, preventing any proton movement.

The nucleotide binding cavity centers on the conserved arginine triplet R84, R183, and R277 which is also a fundamental substrate contact region in other mitochondrial carriers [43]. The triphosphate groups of GTP interact strongly with these residues to anchor the inhibitor at the core of the cavity. Further stabilization is provided by interactions involving Q85, K138, and an important cation pi interaction between R92 and the guanine ring. These extensive contacts restrict the movement of multiple helices and block the conformational change required for proton translocation. Previous studies showing that purine nucleotides greatly stabilize UCP1 are consistent with this structural mechanism [48].

In contrast, fatty acids activate proton conductance and destabilize UCP1 [49]. The fatty acid activation site overlaps with the nucleotide binding region. The anionic headgroup of a fatty acid can interact with the arginine triplet, while hydrophobic residues such as V128, I187, and L278 accommodate the acyl chain. A key residue, D28, forms an ionic interaction with R277 and is essential for proton translocation. Fatty acid binding appears to disrupt the interaction between R277 and D28, loosening the matrix gate and allowing the protein to progress toward a matrix open state. This model aligns with earlier biochemical proposals in which fatty acids function as protonatable cofactors or as transport substrates that support net proton movement [50,51]. It is important to note, however, that no cryo EM or X ray structure of UCP1 bound to a fatty acid currently exists. Therefore, the proposed fatty acid binding mode is not derived from direct structural evidence but instead represents a convergence of functional biochemical and evolutionary data that consistently point to the central cavity and the arginine triplet as the most plausible interaction site.

Accordingly, fatty acid and small-molecule binding modes inferred from molecular docking should be interpreted as hypothesis-based models rather than experimentally validated interactions, and docking scores primarily indicate relative binding plausibility rather than quantitative affinity.

**Figure 3:**
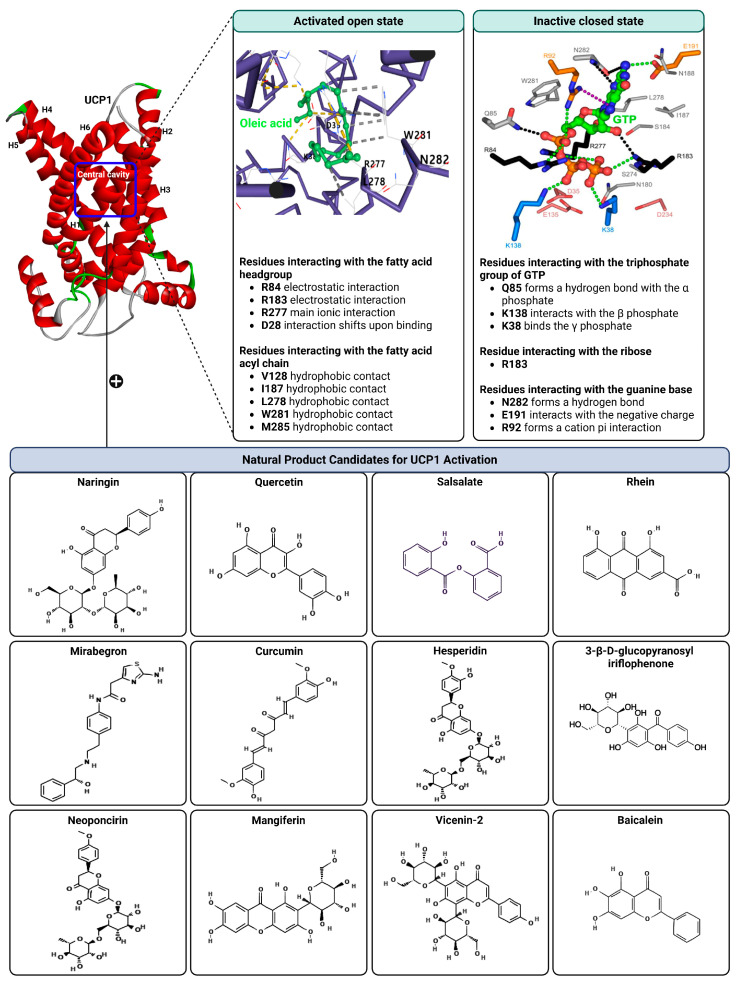
Structural comparison of fatty acid and GTP binding modes in UCP1 and natural product candidates predicted to activate UCP1. The overall structure of human UCP1 is shown on the left, based on the cryo-EM model of the nucleotide-inhibited state (PDB entry 8G8W), which was used for structural comparison. Docking analyses presented in this figure were performed using the ligand-free UCP1 structure (PDB entry 8J1N), and the use of different PDB entries reflects their distinct experimental contexts. The blue box indicates the central cavity of UCP1. The central panel illustrates a predicted docking pose of oleic acid, generated using the CB-Dock2 server (https://cadd.labshare.cn/cb-dock2/ (accessed on 14 October 2025)). In this model, the fatty acid headgroup interacts with the arginine triplet (R84, R183, R277), with R277 serving as the major ionic anchor, while D28 undergoes a positional shift upon ligand engagement. The hydrophobic acyl chain is accommodated within a pocket formed by V128, I187, L278, W281, and M285, consistent with an activated open-state conformation. The right panel depicts the GTP binding mode obtained from the structure reported in reference [43]. The triphosphate group interacts with R84 and Q85 (α phosphate), K138 (β phosphate), and K38, R183, and R277 (γ phosphate), whereas R183 contacts the ribose and N282, E191, and R92 associate with the guanine base. These structural comparisons highlight that fatty acids and nucleotides occupy overlapping regions within the central cavity, supporting their opposing regulatory roles in proton conductance. The bottom panel presents chemical structures of natural product candidates for UCP1 activation, including naringin, quercetin, salsalate, rhein, mirabegron, curcumin, hesperidin, 3-β-D-glucopyranosyl iriflophenone, neoponcirin, mangiferin, vicenin-2, and baicalein. These compounds were selected based on computational or experimental evidence indicating their ability to bind within the UCP1 central cavity or enhance UCP1-mediated thermogenesis. Their structural diversity including flavonoids, benzophenone derivatives, and polyphenolic glycosides reflects the broad chemical space capable of interacting with UCP1 and supports ongoing efforts to identify natural thermogenic activators. All interactions shown are based on in silico predictions and do not represent experimentally validated binding. The cartoon in Figure 3 was created with BioRender.com (https://app.biorender.com, accessed on 15 October 2025).

Overall, the structural findings show that nucleotide inhibition and fatty acid activation involve direct competition for the same central cavity and exert opposing effects on UCP1 conformational flexibility. Nucleotides lock UCP1 in a nonproductive conformation, while fatty acids initiate the transitions required for proton conduction. This framework demonstrates that UCP1 adapts the classical mitochondrial carrier mechanism to fulfill its specialized thermogenic function. The structural features of UCP1, along with its activated and inhibited conformational states, are presented in Figure 3.

## 6. Natural Products as Putative Direct Activators of UCP1

A recent computational study provided important insights into how small molecules interact with human UCP1 at the structural level, offering hypothesis-generating evidence that complements existing experimental observations and expands the framework for UCP1-targeted drug discovery. Using the cryo-EM structure of human UCP1 (PDB ID 8J1N), the authors performed molecular docking, MM-GBSA binding-energy estimation, and 100 ns molecular dynamics simulations to systematically evaluate known UCP1-activating natural compounds and synthetic agents [52].

Several compounds, including naringin, quercetin, salsalate, rhein, mirabegron, curcumin, and formoterol, were predicted to occupy the central cavity of UCP1 with favorable binding characteristics relative to reference uncouplers. The transcriptional and thermogenic effects of quercetin and curcumin are discussed in Section 4. Detailed ligand–residue interaction maps, docking scores, and molecular dynamics stability metrics are provided in Supplementary Table S1. These findings suggest that natural compounds previously associated with enhanced UCP1 expression may also directly engage the canonical DNP or fatty-acid binding cavity in silico, providing a structural rationale for potential direct activation that awaits experimental validation.

Building on these computational insights, a recent in vivo and in silico study reported that a polyphenol-enriched fraction of Cyclopia intermedia increased UCP1 protein levels in brown adipose tissue of obese diabetic mice [53]. Docking analyses further suggested that selected honeybush-derived polyphenols may interact with UCP1. Ligand-specific docking poses and interaction details are summarized in Supplementary Table S1. While these observations support a possible contribution of direct UCP1 engagement, they do not establish a causal relationship between predicted binding and thermogenic activation. Similarly, molecular docking analyses indicated that baicalein may interact with UCP1 in silico [54]. Detailed docking parameters and interaction profiles are provided in the Supplementary Materials. Representative natural products predicted to directly engage UCP1 are summarized in Figure 3.

## 7. Cancer Metabolic Reprogramming and Natural Products Targeting Each Pathway

Tumor cells undergo extensive metabolic reprogramming to sustain rapid growth, resist stress and adapt to nutrient-limited microenvironments. This remodeling involves enhanced glycolysis, altered mitochondrial function, rewired lipid biosynthesis, increased nucleotide production and a heavy reliance on amino-acid-driven anaplerosis [55,56,57]. Several natural products described above as UCP1 modulators, including berberine, curcumin, and resveratrol, exhibit dual metabolic actions by not only enhancing thermogenic programming in adipocytes but also directly reprogramming tumor metabolism. This duality suggests that the same compound can simultaneously increase systemic energy expenditure through UCP1 dependent pathways while restricting nutrient utilization within cancer cells. Building on this concept, the following section summarizes how key natural products influence tumor intrinsic metabolism, with a focus on glycolysis, mitochondrial oxidative phosphorylation, lipid synthesis, and amino acid driven anaplerosis. By integrating these tumor-directed mechanisms with their thermogenic effects in adipose tissue, we highlight how natural compounds may exert coordinated metabolic pressure on cancer progression.

### 7.1. Enhanced Glucose Uptake and Aerobic Glycolysis

Cancer cells substantially increase glucose uptake and convert glucose to lactate even under aerobic conditions, a phenomenon known as the Warburg effect, which provides rapid ATP production and biosynthetic intermediates while contributing to microenvironmental acidification and tumor aggressiveness [58]. Multiple natural products demonstrate experimentally confirmed inhibitory effects on this glycolytic phenotype. p-Synephrine reduces galectin-3–dependent AKT/ERK activation in esophageal squamous cell carcinoma (ESCC) cells, thereby suppressing signaling pathways that support glycolysis and tumor growth [59]. Capsaicin directly down-regulates HK2 expression and decreases glucose uptake and lactate production in ESCC cells [60]. Quercetin inhibits glycolysis by reducing glucose uptake and suppressing GLUT1, HK2 and PKM2 expression in hepatocellular carcinoma, breast cancer and oral squamous cell carcinoma models [61,62,63]. Curcumin attenuates aerobic glycolysis by down-regulating PKM2 through mTOR–HIF-1α inhibition, leading to reduced glucose consumption and lactate production in multiple cancer models [64]. Resveratrol further disrupts glycolytic metabolism by reducing GLUT1, PFK1, PKM2 and LDH expression and lowering lactate output [65]. Additional natural products including naringin, hesperidin and rhein also suppress glycolysis by inhibiting HIF-1α–dependent transcription, down-regulating HK2 and LDHA, or decreasing glucose transport and lactate formation [66,67,68,69,70,71,72,73]. Baicalein inhibits hypoxia-induced HIF-1α expression and its downstream glycolytic targets in ESCC [74], while mangiferin suppresses aerobic glycolysis and glycolysis-associated PI3K/AKT/mTOR signaling through targeting of PFKFB3 in cancer cells [75,76]. These findings collectively demonstrate that many natural compounds interfere with glucose transport, glycolytic enzyme expression or glycolysis-associated transcriptional responses.

### 7.2. Pyruvate Utilization and TCA Cycle Flexibility

Cancer cells typically reduce the entry of pyruvate into mitochondria and instead redirect carbon toward lactate production or cytosolic biosynthetic pathways, resulting in a TCA cycle that functions in a flexible and partially rewired manner optimized for anabolic demands rather than maximal oxidative ATP production. Through the export of citrate for lipid synthesis and the diversion of intermediates such as α-KG and oxaloacetate into biosynthetic and epigenetic pathways, the TCA cycle in tumors primarily operates as a supply route for macromolecule precursors rather than a closed oxidative loop [77]. Several natural products have been shown to interfere with this mitochondria-centered metabolic configuration. Resveratrol enhances mitochondrial pyruvate oxidation by activating the pyruvate dehydrogenase (PDH) complex, thereby counteracting the glycolytic bias of the Warburg phenotype and increasing mitochondrial respiration in colon cancer cells [78]. In contrast, capsaicin impairs mitochondrial complex I and III activity, promoting ROS generation and mitochondrial membrane-potential loss in pancreatic cancer cells [79], while curcumin reduces mitochondrial DNA content through POLG depletion and suppresses oxidative phosphorylation in gastric cancer cells [80]. Berberine further decreases ATP levels and activates AMPK in pancreatic cancer cells, indicating a shift in cellular energy balance away from anabolic support [81]. Collectively, these findings demonstrate that multiple natural products modulate mitochondrial bioenergetics and disrupt the metabolic flexibility that enables tumor cells to dynamically reconfigure pyruvate routing and TCA cycle function, although their precise effects on TCA rewiring have not been directly mapped in the cited studies.

### 7.3. Lipid Biosynthesis Driven by Citrate Export

Citrate exported from mitochondria supplies cytosolic acetyl-CoA for de novo lipid synthesis, supporting membrane biogenesis, redox control and oncogenic lipid signaling. FASN and SREBP-mediated pathways are frequently upregulated across cancer types and contribute to tumor progression [82]. Among the natural products evaluated here, resveratrol is the best characterized for its effects on lipid metabolism. It suppresses de novo lipogenesis, fatty-acid desaturation and cholesterol biosynthesis, thereby limiting membrane-lipid availability and lipid-raft–associated oncogenic signaling [83]. Other compounds such as berberine, PMQ and salsalate activate AMPK in their respective models [68,81,84], a pathway known to inhibit lipogenesis; however, the specific effects of these compounds on cancer-cell lipid metabolism were not measured directly in the cited studies. Reviews summarizing the broader anticancer metabolic activity of some agents, including rhein and hesperidin, also describe potential interactions with lipid-associated pathways, although these were not tested experimentally in the included primary studies [85,86,87]. Thus, resveratrol remains the principal natural compound with direct experimental evidence of disrupting tumor lipid biosynthesis in this dataset.

### 7.4. Increased Nucleotide Biosynthesis

Rapid tumor proliferation requires sustained de novo nucleotide synthesis, fueled by glycolytic intermediates, TCA cycle-derived carbon skeletons and pentose phosphate pathway activity, making cancer cells highly sensitive to perturbations in nucleic-acid precursor supply [88]. Within the natural products covered in this review, only a subset has been directly tested for effects on nucleotide-synthesis enzymes or DNA precursor flux in cancer models. Berberine interferes with the folate cycle in cisplatin-sensitive and -resistant ovarian cancer cells by down-regulating dihydrofolate reductase (DHFR) and thymidylate synthase (TS) expression, thereby impairing folate-dependent one-carbon transfer reactions that are essential for de novo thymidylate and purine biosynthesis [89]. Resveratrol acts as a potent inhibitor of ribonucleotide reductase in mammalian tumor cells, reducing deoxyribonucleotide production and DNA synthesis, which provides a direct link between this polyphenol and suppression of de novo dNTP generation [90]. Quercetin has been shown to enhance 5-fluorouracil efficacy in colorectal cancer cells by preventing 5-FU-induced upregulation of TS and by lowering TS protein levels in both in vitro and in vivo models, thereby undermining a key resistance mechanism and functionally constraining thymidylate production [91].

### 7.5. Glutamine Addiction and Anaplerosis

Glutamine functions as a central carbon and nitrogen donor in many cancer types, sustaining TCA cycle anaplerosis, glutathione biosynthesis and α-KG–dependent epigenetic regulation. Tumors driven by MYC are particularly dependent on glutamine metabolism and frequently exhibit a “glutamine-addicted” phenotype [92].

Recent findings indicate that some natural products can directly interfere with glutamine utilization in cancer cells. Curcumin has been shown to suppress glutamine metabolism in cisplatin-resistant colon cancer cells by up-regulating microRNA-137, which targets and inhibits glutaminase (GLS), thereby reducing glutamine uptake and GLS enzymatic activity [93]. In hepatocellular carcinoma, berberine decreases glutamine dependence by down-regulating the glutamine transporter SLC1A5, resulting in reduced glutamine uptake and impaired tumor growth in vitro and in vivo [94]. In gemcitabine-resistant cholangiocarcinoma, curcumin further inhibits glutamine utilization by suppressing L type amino acid transporter 2 (LAT2)-mediated glutamine transport and reducing the expression of glutaminase and glutamine synthetase, thereby enhancing chemosensitivity [95].

For the remaining natural products analyzed in this review none of the cited studies provided direct experimental evidence of inhibition of glutamine uptake, glutaminase activity, α-KG generation or glutamine-derived anaplerosis. Although some of these compounds modulate mitochondrial bioenergetics or activate AMPK, the available data do not demonstrate a measured effect on glutamine metabolism within the referenced experimental systems.

### 7.6. ATP Production: Oxidative and Non-Oxidative Contributions

**Figure 4:**
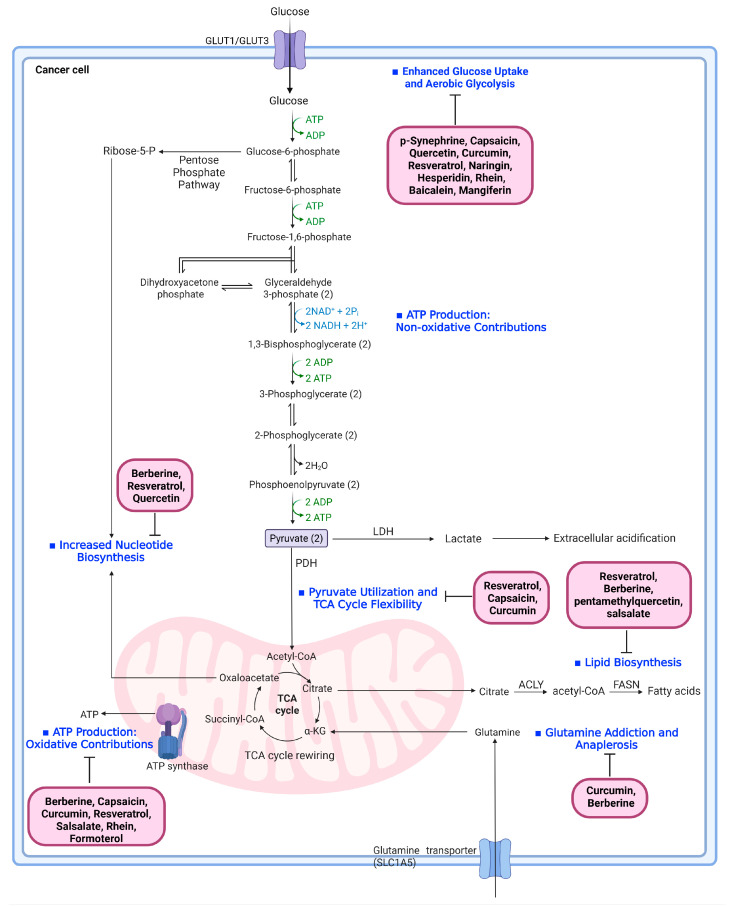
Natural product–mediated modulation of cancer metabolic reprogramming. This figure summarizes the major metabolic pathways reprogrammed in cancer cells and highlights the experimentally validated sites at which natural products exert inhibitory effects. Enhanced glucose uptake and aerobic glycolysis are disrupted by multiple compounds, including p-synephrine, capsaicin, quercetin, curcumin, resveratrol, naringin, hesperidin, rhein, baicalein and mangiferin. Across representative preclinical studies, these interventions reduce glucose uptake or glycolytic flux by approximately ~30–70%, depending on cell type and experimental context. Nucleotide biosynthesis is affected by berberine, resveratrol and quercetin through inhibition of folate-cycle enzymes, ribonucleotide reductase or thymidylate synthase. Pyruvate utilization and TCA cycle flexibility are modulated by resveratrol, capsaicin and curcumin via activation of PDH, inhibition of mitochondrial electron-transport complexes or suppression of oxidative phosphorylation. These effects are commonly associated with an approximate ~20–50% reduction in cellular ATP production. ATP production from oxidative and non-oxidative metabolism is regulated by berberine, capsaicin, curcumin, resveratrol, salsalate, rhein and formoterol. Lipid biosynthesis downstream of citrate export is suppressed by resveratrol, berberine, PMQ and salsalate. Curcumin and berberine additionally inhibit glutamine addiction and anaplerosis by targeting glutaminase activity, glutamine transport or LAT2-dependent uptake. Collectively, the diagram illustrates how diverse natural compounds interfere with metabolic circuits that support tumor growth and survival. The cartoon in Figure 4 was created with BioRender.com (https://app.biorender.com, accessed on 8 November 2025). Abbreviations: PDH, pyruvate dehydrogenase; TCA cycle, tricarboxylic acid cycle; PMQ, pentamethylquercetin; LAT2, L-type amino acid transporter 2.

Cancer cells generate ATP through both glycolysis and oxidative phosphorylation, with metabolic plasticity allowing dynamic adaptation to oncogenic signals and nutrient availability [96]. Several natural products influence cellular ATP homeostasis through validated metabolic mechanisms. Berberine reduces ATP levels and activates AMPK in pancreatic cancer cells by inducing mitochondrial depolarization [81]. Capsaicin diminishes mitochondrial membrane potential and promotes ROS-mediated dysfunction, affecting ATP generation [79]. Curcumin decreases mitochondrial DNA content and suppresses oxidative phosphorylation, resulting in reduced ATP output [80]. Resveratrol enhances PDH activity and mitochondrial respiration while reducing glycolytic ATP production, thereby shifting metabolic ATP origins [78]. Salsalate activates AMPK via direct salicylate binding [68] and modifies glucose-depletion metabolic stress responses in lung adenocarcinoma cells [69]. Rhein lowers oxygen consumption and lactate production in Ehrlich ascites tumor cells by inhibiting glucose uptake [70,71], while formoterol modulates oxidative stress and ATP-related metabolic disruptions in smoke-exposed lung adenocarcinoma cells [97]. These findings highlight that diverse natural compounds can influence ATP production by acting on glycolysis, mitochondrial function or energy-stress signaling pathways. A summary of bioactive compounds and their metabolic regulatory actions in cancer cells is presented in Table 2 and Figure 4.

## 8. Research Gaps and Future Directions

Despite substantial advances in UCP1 biology and natural product pharmacology, these areas have not yet been integrated into a unified experimental framework relevant to cancer metabolism. Accordingly, the overarching objective of future research is to determine whether natural product-induced activation of UCP1-dependent thermogenesis can causally suppress tumor growth through metabolic nutrient competition. This objective addresses a central unmet question in the field by explicitly linking adipocyte thermogenic programming, systemic energy expenditure, and tumor metabolic vulnerability within a single testable framework.

### 8.1. Existing Evidence Linking Natural Products, Adipose Biology, and Tumor Regulation

Recent work has demonstrated that metabolically engineered adipocytes can function as active metabolic competitors against tumors. Nguyen et al. showed that CRISPR activation of *UCP1*, *PPARGC1A* and *PRDM16* converts human white adipocytes into highly oxidative beige-like cells that consume glucose and fatty acids at elevated rates; when co-cultured or co-implanted with cancer cells, these engineered adipose organoids suppress tumor glycolysis and growth through nutrient competition [8]. In parallel, several natural products have been shown to modulate adipocyte biology in ways that influence tumor behavior, although none of these studies directly employ UCP1-dependent thermogenesis. Piceatannol inhibits cancer-induced lipolysis in 3T3-L1 adipocytes and attenuates adipose wasting in cancer-cachexia models [101]; PMQ reduces hepatocellular carcinoma progression by altering adipocyte differentiation and decreasing adipocyte-derived IFN-γ, which in turn lowers PD-L1 expression in HepG2 cells [102]; curcumin disrupts CXCL12/CXCR4-mediated feedback between ADMSCs and MCF-7 cells, thereby reducing stromal activation and EMT [103]; and dasatinib plus quercetin reduces adipose-tissue metastasis in ovarian cancer by targeting senescent adipose-derived stem cells [104]. These studies collectively illustrate that natural products can modulate adipocyte function or adipose-derived stromal cells in cancer-relevant contexts.

### 8.2. Critical Mechanistic Gap in Natural-Product-Driven Thermogenic Competition

As outlined in the Introduction, the complete mechanistic sequence linking natural product–induced UCP1 thermogenesis to tumor suppression via nutrient competition has not yet been experimentally established [77,105]. Existing studies typically address only individual components of this pathway, either focusing on beige [6,106] or brown adipocyte induction in metabolic disease contexts or examining tumor-intrinsic metabolic suppression by natural products [6,106]. These lines of investigation have largely proceeded in parallel, without integrating adipocyte-driven thermogenic activation and direct competition for metabolic substrates within a unified experimental framework. Even studies exploring adipocyte–cancer interactions predominantly emphasize endocrine or paracrine remodeling effects, rather than demonstrating a causal role for UCP1-dependent thermogenesis in limiting tumor nutrient uptake. Consequently, whether pharmacological induction of UCP1-high thermogenic adipocytes by natural products can function as a bona fide metabolic sink to deprive tumors of glucose and lipids remains unresolved. Based on this gap, it is hypothesized that pharmacological activation of UCP1 in adipocytes by selected natural products generates a thermogenic metabolic sink that competitively restricts tumor access to essential nutrients, thereby suppressing tumor growth.

### 8.3. Proposed Experimental Strategies to Bridge the Gap

To address this gap, future work should systematically evaluate natural products known to influence both adipocyte function and tumor metabolism such as curcumin, quercetin derivatives, berberine, piceatannol/resveratrol analogues and PMQ or their ability to generate bona fide beige/brown adipocytes [107]. This includes quantifying UCP1, PGC-1α and PRDM16 expression, mitochondrial biogenesis, oxygen-consumption rate and thermogenic competence in white adipocytes or adipose organoids after chronic exposure to each compound. Natural-product-treated adipocytes should then be incorporated into co-culture systems with cancer cells to determine whether increased adipocyte substrate oxidation reduces tumor glucose/fatty-acid uptake, glycolytic flux, ATP production and proliferation. Integration of metabolic assays, nutrient consumption, extracellular acidification, oxygen consumption, ATP measurement and transcriptional profiling, will be essential for demonstrating nutrient competition analogous to that seen in engineered AMT models. Promising candidates should finally be validated in vivo using tumor-bearing mice, with careful distinction between systemic metabolic effects and local nutrient competition, ideally using metabolic tracers and adipose-specific UCP1 loss-of-function approaches [15,108].

To disentangle systemic metabolic effects from tumor-intrinsic responses, future studies should incorporate adipose-specific Ucp1 knockout models alongside wild-type controls, or utilize implantation of engineered beige adipocytes with defined thermogenic capacity. In parallel, tumor-intrinsic metabolic fluxes should be directly quantified using stable isotope tracer approaches, such as ^13^C-labeled glucose, palmitate, or glutamine, to assess competitive nutrient utilization between thermogenic adipose tissue and tumors in vivo. Such experimental designs would allow causal attribution of tumor growth suppression to adipose-driven UCP1-dependent thermogenesis rather than secondary systemic or tumor-cell–autonomous effects [109].

Collectively, these approaches provide a clear experimental path to test the central hypothesis and to establish objective, mechanism-based evidence for natural product–driven UCP1 thermogenic competition in cancer.

## 9. Conclusions

This review synthesizes two previously separate domains: natural products that promote adipocyte browning and UCP1 activation, and natural compounds that suppress tumor metabolism. By identifying that these domains have not yet been experimentally integrated, the review highlights a precise and testable research gap. The natural-product-driven, UCP1-dependent thermogenic adipocyte model proposed here thus represents a novel conceptual and experimental framework. Future studies based on this model could yield an entirely new class of metabolic anticancer strategies that exploit competition for glucose and fatty acids at the adipocyte–tumor interface.

Despite the conceptual appeal of UCP1-centered thermogenic competition, several translational challenges must be addressed before clinical application can be considered. Many natural products induce thermogenic responses at micromolar concentrations in vitro, which may exceed achievable plasma levels in humans due to limited absorption, rapid metabolism, and suboptimal tissue distribution. Overcoming these pharmacokinetic and bioavailability constraints may require formulation strategies such as nanoparticle delivery systems, prodrug approaches, or chemically modified analogs, including methylated derivatives such as pentamethylquercetin.

In addition, systemic activation of thermogenesis carries potential safety risks, including excessive weight loss, hypermetabolism, cardiovascular strain, and disrupted glucose homeostasis. These effects may be particularly relevant in vulnerable patient populations, such as individuals with metabolic disorders, advanced malignancy-associated cachexia, or underlying cardiovascular disease. Careful patient selection, clearly defined exclusion criteria, and prospective monitoring of metabolic, cardiovascular, and nutritional parameters will therefore be essential in early-phase translational studies.

Finally, many bioactive natural compounds, including polyphenols and alkaloids, are known to modulate cytochrome P450 enzymes and drug transporters, raising the possibility of clinically relevant drug–drug interactions. This issue is especially pertinent in oncology settings, where polypharmacy is common. Rigorous preclinical interaction screening, coupled with prospective clinical pharmacovigilance, will be required to ensure the safe integration of thermogenic natural products with standard anticancer therapies.

## Figures and Tables

**Table 1:** Natural Products That Upregulate UCP1.

Natural Product	Cells/Tissues	Dose Concentration	UCP1-Related Findings	Ref.
Natural β-adrenergic mimetics				
p-Synephrine 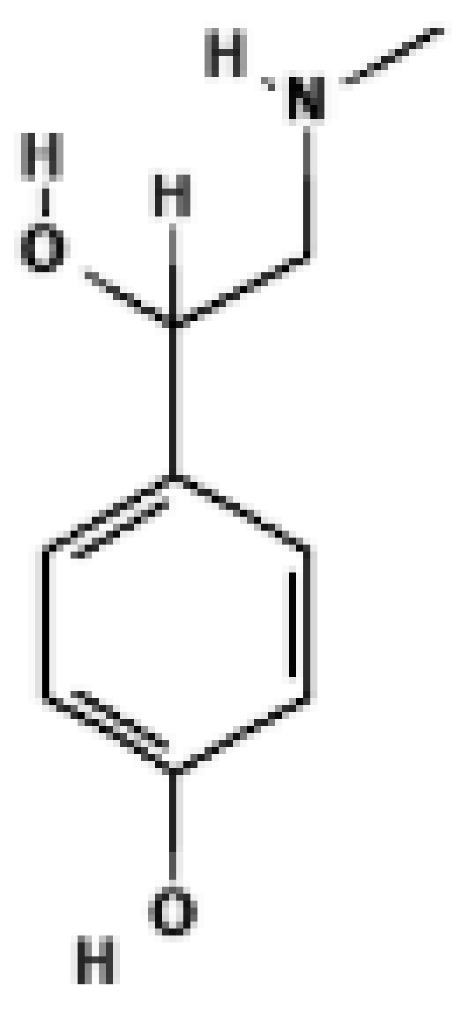	Mouse SVF-derived beige adipocytes	3.12–12.5 μM	↑ UCP1 mRNA in a dose-dependent manner; induces beige morphology; effect abolished by β3-AR antagonist	[31]
Capsaicin 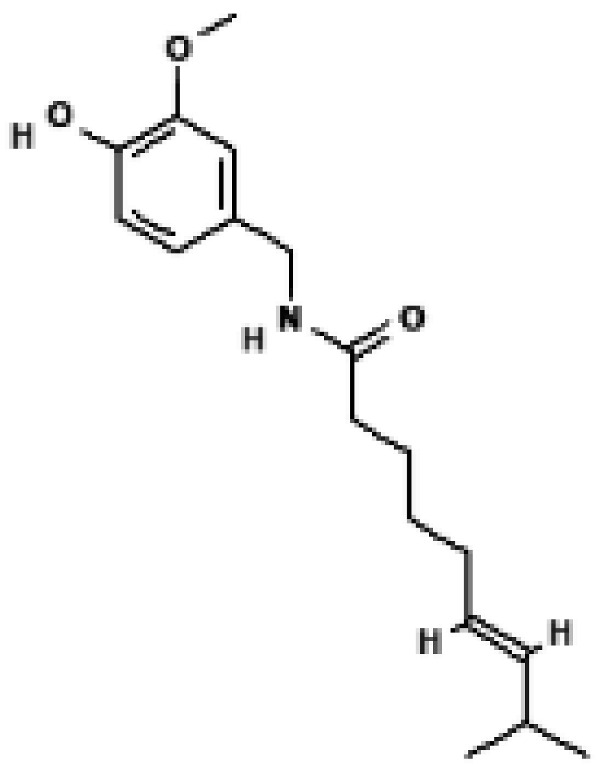	Primary WAT preadipocytes, EF/SCF (mouse)	0.1–10 μM (cells), 0.01% diet (mice)	TRPV1-dependent Ca^2+^ influx → CaMKII/AMPK/SIRT1 activation → ↑ UCP1, ↑ PRDM16, ↑ PGC1α → WAT browning and anti-obesity effect	[32]
Capsinoid 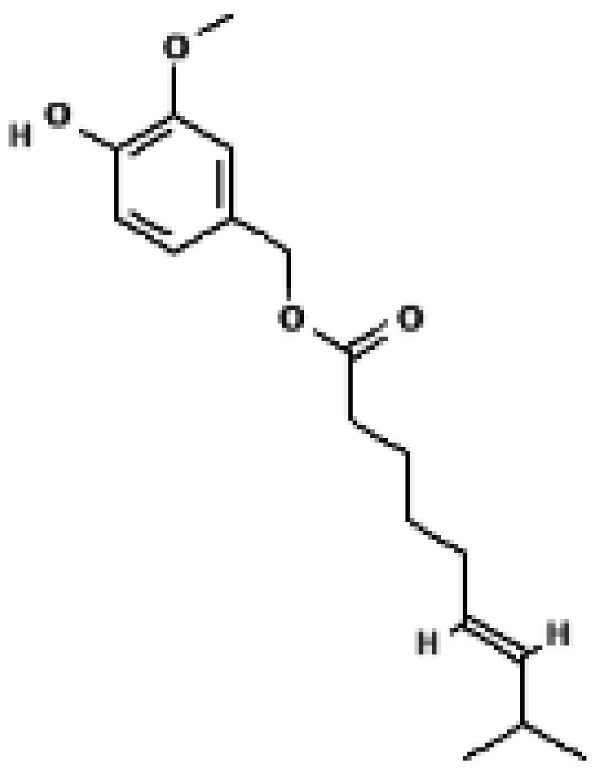	Human brown adipose tissue (FDG-PET)	9 mg/day (oral ingestion)	↑ Whole-body energy expenditure → ↑ Cold-induced BAT activation (FDG uptake) → Activation of UCP1 positive depots	[33]
AMPK-activating natural products				
Berberine 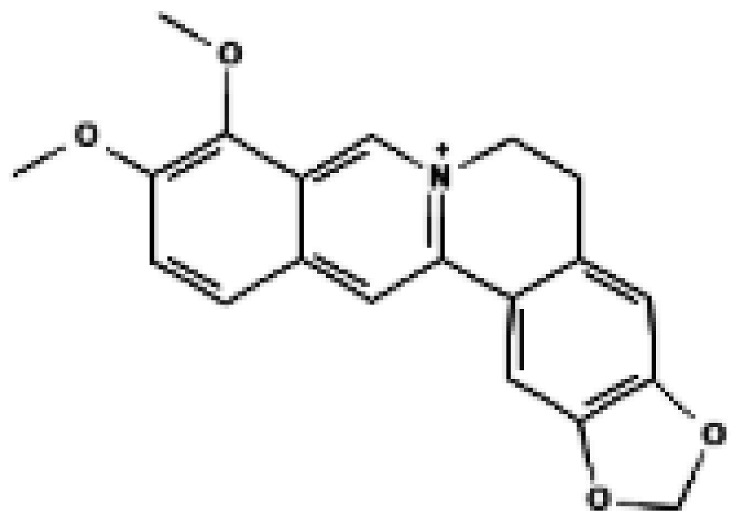	Primary brown adipocytes, primary inguinal white adipocytes, C3H10T1/2 adipocytes, BAT, iWAT	0.5 μM, 2.5 μM (in vitro); 5 mg/kg/day i.p., 4 weeks (in vivo)	↑ UCP1 mRNA/protein, ↑ PGC1α, ↑ PRDM16, WAT browning, ↑ oxygen consumption, ↑ whole-body thermogenesis	[34]
	Human NAFLD patients (BAT), Mouse BAT, iWAT, eWAT, Mouse & human primary brown preadipocytes, Mouse BAT-SVF and C3H10-T1/2 cells	In vitro: 0.25~2 μM (dose-dependent), In vivo (mouse): 1.5 mg/kg/day i.p. for 6 weeks, In humans: 0.5 g orally, 3×/day for 1 month	↑PRDM16 → ↑ UCP1 mRNA and protein in mouse and human brown adipocytes → ↑ Brown adipocyte differentiation → ↑ BAT mass & thermogenic activity	[35]
Quercetin 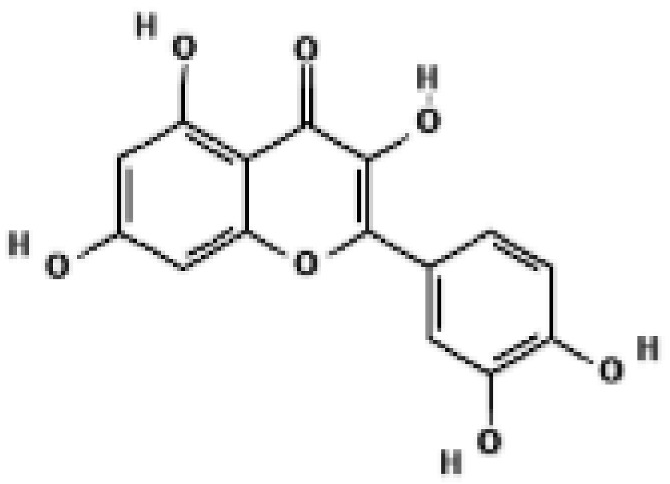	WAT, BAT; 3T3-L1	40 μg/mL	↑ UCP1, ↑ AMPK	[36]
Ginsenoside Rg1 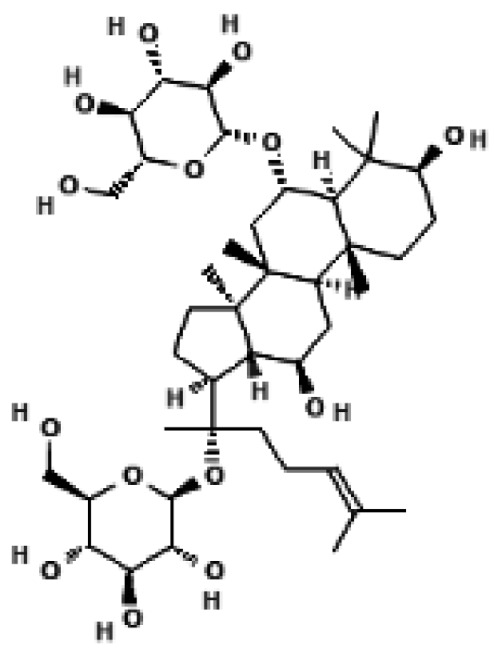	3T3-L1 adipocytes; mouse subcutaneous white adipocytes (scWAT)	25, 50, 100 μM (dose-dependent), 50 μM used for main experiments	↑ PRDM16, ↑ PGC1α, ↑ UCP1 protein and mRNA in a dose-dependent manner → promotes adipocyte browning and mitochondrial biogenesis via AMPK activation	[37]
Black ginseng extract; Ginsenoside Rb1 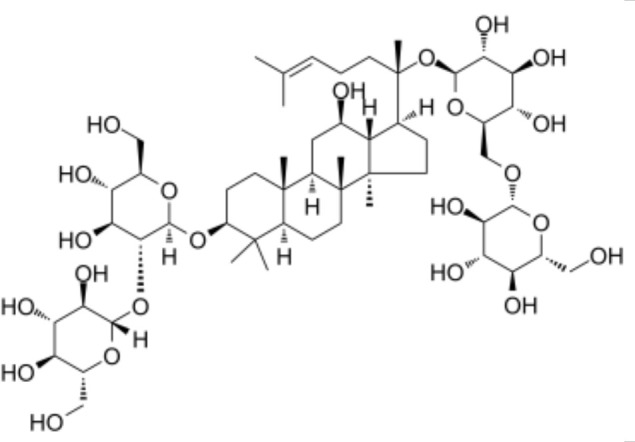	3T3-L1 adipocytes; Primary white adipocytes (PWATs)	10, 20, 40 µM	↑ UCP1, ↑ PRDM16, ↑ PGC1α, ↑ p-AMPK → promotes browning of 3T3-L1 and PWATs	[38]
Curcumin 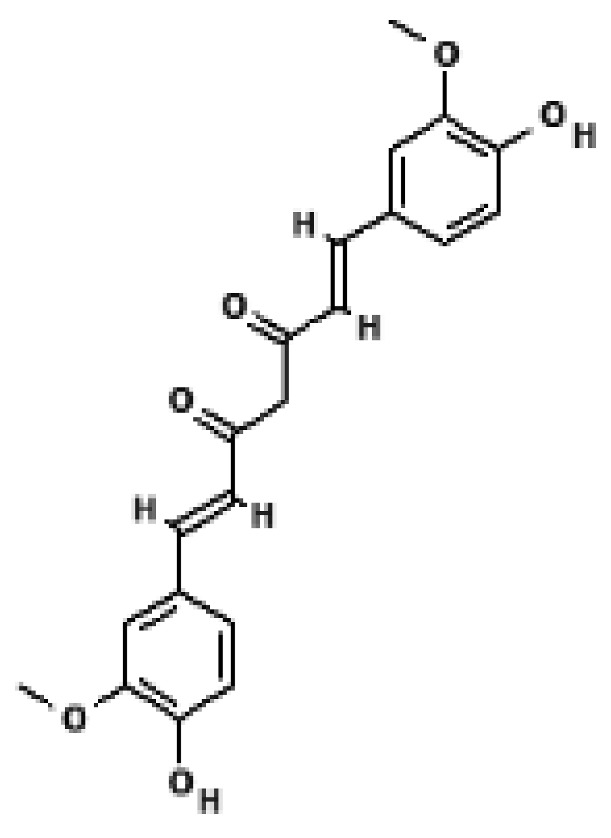	3T3-L1 adipocytes; primary white adipocytes	1–20 μM	↑ UCP1, ↑ PRDM16, ↑ PGC1α, ↑ mitochondrial biogenesis; AMPK-dependent browning response	[39]
	C57BL/6J mice (BAT, WAT), RAW264.7 macrophages, rat primary adipocytes, mBAC brown adipocytes	In vitro: 0.25–20 μM (RAW264.7, adipocytes, mBAC) In vivo: 1% dietary curcumin in HFD	↑ UCP1 mRNA & protein in BAT, ↑ UCP1 promoter activity (PPARα/γ-dependent & independent)	[40]
PRDM16-enhancing natural products				
Berberine	Human NAFLD patients (BAT), Mouse BAT, iWAT, eWAT, Mouse & human primary brown preadipocytes, Mouse BAT-SVF and C3H10-T1/2 cells	In vitro: 0.25~2 μM (dose-dependent), In vivo (mouse): 1.5 mg/kg/day i.p. for 6 weeks, In humans: 0.5 g orally, 3×/day for 1 month	↑ PRDM16, ↑ UCP1	[35]
Curcumin	3T3-L1 adipocytes; primary white adipocytes	1–20 μM	↑ PRDM16, ↑ PGC1α, ↑ UCP1	[39]
Ginsenoside Rg1	3T3-L1 adipocytes; mouse subcutaneous white adipocytes (scWAT)	25, 50, 100 μM (dose-dependent), 50 μM used for main experiments	↑ PRDM16, ↑ UCP1	[37]
Ginsenoside Rb1	3T3-L1 adipocytes; Primary white adipocytes (PWATs)	10, 20, 40 µM	↑ PRDM16	[38]
Mitochondrial biogenesis enhancers				
Resveratrol 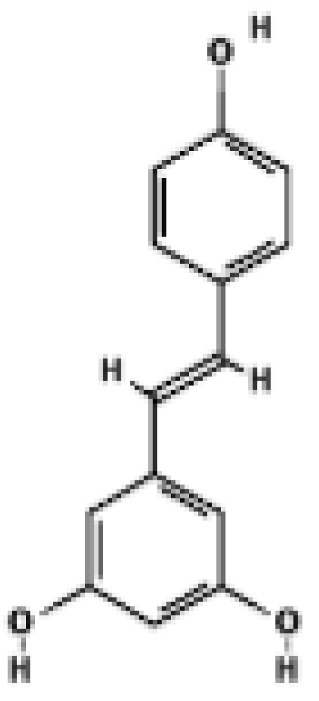	C57BL/6J mice BAT	200–400 mg/kg/day, 15 weeks (diet) Plasma RSV 10–120 ng/mL	↑ Mitochondrial size & cristae density in BAT and muscle ↑ mtDNA content (BAT & muscle) ↑ UCP1 mRNA in BAT	[41]
PMQ 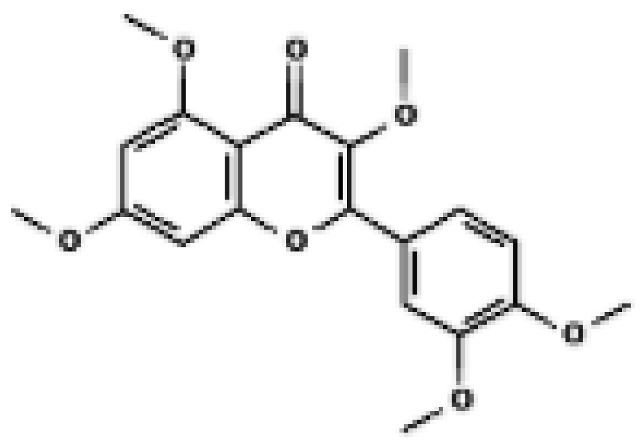	3T3-L1 adipocytes; Epididymal WAT & BAT of HFD mice	In vitro: 0.1–10 µM (most effects at 10 µM) In vivo: HFD + 0.04% PMQ diet (≈40 mg/kg/day)	↑ Mitochondrial markers (Cytochrome C) in WAT ↑ UCP1-positive multilocular adipocytes in WAT of HFD mice	[42]

**Table 2:** Natural products and their inhibitory effects on cancer cell metabolism.

Natural Product	Metabolic Pathway Inhibited	Key Targets/Mechanisms	Ref.
p-Synephrine	Indirect inhibition of glycolysis via suppression of metabolic signaling	↓ Galectin-3–mediated activation of AKT and ERK; inhibition of downstream glycolysis-supportive signaling pathways	[59]
Capsaicin	Inhibition of aerobic glycolysis and mitochondrial electron transport chain (ETC)	↓ HK2, ↓ glucose uptake, ↓ lactate; inhibition of mitochondrial complexes I & III → ROS-mediated apoptosis	[60,79]
Berberine	Inhibition of glycolysis and oxidative phosphorylation (OXPHOS)	Mitochondrial depolarization → AMPK activation; ↓ ATP; impaired glycolytic capacity and mitochondrial function	[81,98]
Quercetin	Inhibition of aerobic glycolysis	↓ HK2, ↓ GLUT1, ↓ PKM2; ↓ Akt–mTOR signaling; ↓ glucose uptake, ↓lactate production	[61,62,63]
Curcumin	Suppression of glycolysis and mitochondrial respiration	↓ PKM2 via mTOR–HIF-1α inhibition; ↓ glucose uptake and lactate; POLG depletion → mitochondrial dysfunction	[64,80]
Resveratrol	Inhibition of glycolysis and lipid synthesis	↓ GLUT1, ↓ PFK1, ↓ PKM2, ↓ LDH; inhibition of PI3K–AKT–mTOR and HIF-1α; activation of PDH and mitochondrial oxidation	[65,78,83]
PMQ	Indirect inhibition of glycolysis-supportive signaling	↑ AMPK → ↓ mTOR → ↓ anabolic/glycolytic signaling	[84]
Naringin	Inhibition of aerobic glycolysis in cancer cells	HIF-1α ↓ → ENO2 ↓ → glycolysis ↓; c-Src phosphorylation ↓ → glucose metabolism ↓	[66,67]
Salsalate	Inhibition of glycolysis-supportive and anabolic metabolism	↑ AMPK → ↓ mTOR/anabolic signals; ↑ fatty-acid oxidation; ↓ de novo lipogenesis	[68,69]
Rhein	Inhibition of glycolysis (glucose uptake → lactate production)	↓ glucose transporter function → ↓ glucose uptake → ↓ lactate production	[70,71,85,86]
Mirabegron	Indirect modulation of tumour metabolism (via adipose browning)	↑ β3-AR → ↑ UCP1 → ↑ thermogenesis → systemic fuel redistribution	[99]
Formoterol	Indirect modulation of tumour-associated metabolism	↑ β_2_-AR → systemic substrate shift (glycolysis/TCA/lipid metabolites altered)	[97,100]
Hesperidin	Inhibition of glycolysis	↓ HK2, ↓ LDHA; ↓ lactate; enhanced chemosensitivity with doxorubicin	[72,73,87]
Mangiferin	Inhibition of aerobic glycolysis	↓ PFKFB3-mediated glycolysis → ↓ PI3K/AKT/mTOR signalling	[75,76]
Baicalein	Inhibition of HIF-1α–regulated glycolysis	↓ HIF-1α → ↓ HIF-1α–controlled glycolytic genes	[74]

## Data Availability

No new data were created or analyzed in this study. Data sharing is not applicable to this article.

## Supplementary Materials

The following supporting information can be downloaded at: https://www.mdpi.com/article/10.3390/biom16010090/s1, Table S1: Natural products that activate UCP1.
