# Supplementary material for: Natural Product Driven Activation of UCP1 and Tumor Metabolic Suppression: Integrating Thermogenic Nutrient Competition with Cancer Metabolic Reprogramming
Source: Biomolecules. 2026 Jan 6;16(1):90. doi: 10.3390/biom16010090 (PMC12839171; doi:10.3390/biom16010090)
Supplement: Supplementary file 1 [file biomolecules-16-00090-s001.zip › biomolecules-4027341-supplementary.pdf]

Supplementary Table S1. Natural products that activate UCP1.

Natural compound	Docking method	Docking score (kcal/mol)	Major interacting residues	Ref.
Naringin 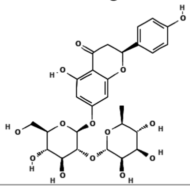	Glide XP (PDB: 8J1N)	−7.284	Asp34, Arg83, Ser87, Arg91, Arg182, Asn187, Glu190	[52]
Quercetin	Glide XP	−6.661	Asp34, Arg83, Glu190, Asn281	[52]
Salsalate 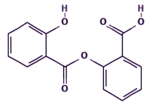	Glide XP	−6.017	Trp280, Arg91, Arg83, Asn281, Glu190	[52]
Rhein 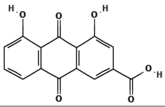	Glide XP	−5.8	Arg83, Arg91, Trp280, Asn281, Glu190	[52]
Mirabegron 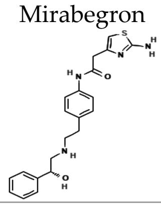	Glide XP	−5.5	Arg83, Arg91, Trp280, Asn281, Glu190	[52]
Curcumin	Glide XP	−5.5	Asn187, Arg276, Trp280, Glu190	[52]
Formoterol 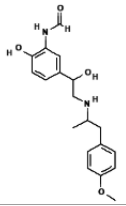	Glide XP	−5.5	Glu190, Arg91, Arg182, Trp280, Asn281	[52]
Hesperidin 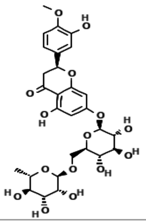	AutoDock Vina (AF-Q9Z2I0-F1)	−8.8	Lys38, Val39, Arg40, Gly45, Gln48, Glu46, Ala143, Gln144, Glu168, Gly176, Asn180, Pro179, Arg183, Phe240, Ile241	[53]
3-β-D-glucopyranosyl iriflophenone 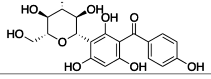	AutoDock Vina	−8.0	Lys38, Val39, Glu46, Gly45, Arg40, Ala143, Phe240; H-bonds: Gln48, Thr36, Arg140	[53]

Neoponcirin 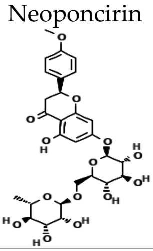	AutoDock Vina	−7.9	Lys350, Val139, Val39, Pro179, Gly47, Phe240, Ile241, Leu244, Lys175, Thr172; H-bonds: Arg183, Arg140, Arg40, Ala143	[53]
Mangiferin 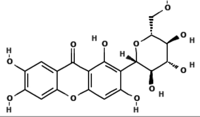	AutoDock Vina	−7.6	Lys38, Val39, Arg40, Gly45, Pro179, Phe240	[53]
Vicenin-2 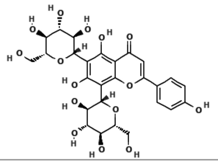	AutoDock Vina	−7.2	Not reported	[53]
Baicalein 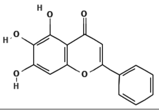	AutoDock Vina	−7.6	Arg239, Asn242, Phe240, Val258, Tyr248, Ser250, Lys257, Glu262, Val251	[54]
